# Neural Correlates of Task Cost for Stance Control with an Additional Motor Task: Phase-Locked Electroencephalogram Responses

**DOI:** 10.1371/journal.pone.0151906

**Published:** 2016-03-24

**Authors:** Ing-Shiou Hwang, Cheng-Ya Huang

**Affiliations:** 1 Department of Physical Therapy, College of Medicine, National Cheng Kung University, Tainan 701, Taiwan; 2 Institute of Allied Health Sciences, College of Medicine, National Cheng Kung University, Tainan 701, Taiwan; 3 School and Graduate Institute of Physical Therapy, College of Medicine, National Taiwan University, Taipei 100, Taiwan; 4 Physical Therapy Center, National Taiwan University Hospital, Taipei 100, Taiwan; Duke University, UNITED STATES

## Abstract

With appropriate reallocation of central resources, the ability to maintain an erect posture is not necessarily degraded by a concurrent motor task. This study investigated the neural control of a particular postural-suprapostural procedure involving brain mechanisms to solve crosstalk between posture and motor subtasks. Participants completed a single posture task and a dual-task while concurrently conducting force-matching and maintaining a tilted stabilometer stance at a target angle. Stabilometer movements and event-related potentials (ERPs) were recorded. The added force-matching task increased the irregularity of postural response rather than the size of postural response prior to force-matching. In addition, the added force-matching task during stabilometer stance led to marked topographic ERP modulation, with greater P2 positivity in the frontal and sensorimotor-parietal areas of the N1-P2 transitional phase and in the sensorimotor-parietal area of the late P2 phase. The time-frequency distribution of the ERP primary principal component revealed that the dual-task condition manifested more pronounced delta (1–4 Hz) and beta (13–35 Hz) synchronizations but suppressed theta activity (4–8 Hz) before force-matching. The dual-task condition also manifested coherent fronto-parietal delta activity in the P2 period. In addition to a decrease in postural regularity, this study reveals spatio-temporal and temporal-spectral reorganizations of ERPs in the fronto-sensorimotor-parietal network due to the added suprapostural motor task. For a particular set of postural-suprapostural task, the behavior and neural data suggest a facilitatory role of autonomous postural response and central resource expansion with increasing interregional interactions for task-shift and planning the motor-suprapostural task.

## Introduction

Human upright stance is thought to be a semiautomatic sensory-motor process of centering the center of mass above the base of support [1,2]. The maintenance of upright stance in daily activities often lays the basis for a suprapostural task with a perception-action goal [3]. On account of the task interference effect, central resources should be reorganized to execute the added task component without loss of stance balance [4]. Previous behavioral studies indicate that, contrary to the parallel loading of two cognitive tasks, the central resources of a postural-cognitive task do not necessarily compete with each other and can be shared by two response programs [5,6]. In the behavior studies, posture-cognition interaction especially for a main effect of a secondary cognitive task on postural task was assessed with irregularity of postural sway [7,8,9,10]. These authors reported that postural sway during dual task performance could became more random with higher entropy values, as a behavior context of increasing autonomous postural control or less attentive resource being paid to the postural component [11]. As dual-task performance varies with the response compatibility of two component tasks, adding a motor task to upright stance could differently challenge resource allocation, similar to the case of a postural-cognitive task [12]. Both posture and secondary motor tasks could compete for a motor-specialized resource, and the brain needs to coordinate reciprocally-related limb dynamics while integrating motor-suprapostural tasks into postural control. Although the issue has been debated for years [13], the way the brain minimizes task costs for upright stance with an additional motor task is still unknown.

Event related potentials (ERPs) are often used to explore the neural mechanism of task organization in a dual-task. In comparison with the oddball-only condition, N140 and P300 amplitudes decrease in the dual-task condition (performing oddball and visuomotor tracking concurrently) [14], and P300 amplitude wanes specifically with increasing difficulty of the secondary visuomotor task [15]. For a visuo-spatial working memory task, enhancement of long-range theta coupling (4–7 Hz or 5–7 Hz) in the fronto-parietal network has been noted to reflect a top-down control process for task-switching between memory systems and execution demand in human [16,17] and monkey [18] experiments. In the concurrent execution of unrelated visual perception and working memory tasks, beta oscillation (18–24 Hz) was enhanced over that in the single-task condition, functionally serving to interface frontal-executive and occipitoparietal-perceptual processes [19]. Our previous work on a posture-motor task also revealed that ERPs in the fronto-parietal networks are important in monitoring of the attentional states in a postural-suprapostural task [20,21]. While maintaining upright stance and performing a force-matching task (a motor-suprapostural task), N1 negativity varied positively with the level of postural instability [20,21]. This N1 negativity of posture-related ERP is thought to originate in the fronto-central regions [22], and to be relayed to anticipatory arousal [23] and sensory processing of postural perturbation [24,25]. On the other hand, P2 positivity was related inversely to the task-load of the secondary force-matching task [20].

Previous ERP-related dual-task studies tended to gravitate toward the binding of cognitive-cognitive or cognitive-motor tasks, which involve two distinct neurological regions defined by their functional roles in cognition. For intensive sharing of sensorimotor resources, the cortical mechanism for a cross-modal postural-motor task could be distinct from that of a classic dual-task situation [3,26]. Also, a simple bottleneck model based on traditional dual-task setups may not be appropriate for a postural-suprapostural task, by virtue of postural prioritization [20,27] and facilitation of suprapostural activities with reduced postural sway [28]. By characterizing N1 and P2 components of ERPs, this study sought to gain better insight into the cortical mechanisms of resource reallocation upon the addition of a motor task on stabilometer stance. Based on resource-competition effects, we hypothesized that 1) compared to a single-posture condition, the postural performance would be impaired when a motor-suprapostural task was added, and 2) resource operation would be reorganized to cope with a motor-suprapostural task. For the reciprocal effect, we expected variations in the topographies and the temporal and spectral features of the N1 and P2 components in the fronto-parietal network between the single-posture and dual-task conditions.

## Materials and Methods

### Subjects

Twelve adults from the university campus (6 males, 6 females; mean age: 22.9 ± 2.0 years) who had normal or corrected-to-normal vision and no neurological or balance disorders participated in the study. They were all right-hand dominant by self-report. All the subjects gave informed consent for the experimental procedure approved by the local Institutional Review Board (National Taiwan University Hospital Research Ethics Committee; no. 201312077RINC) to protect the rights of human subjects.

### Experimental procedures

**Fig 1:**
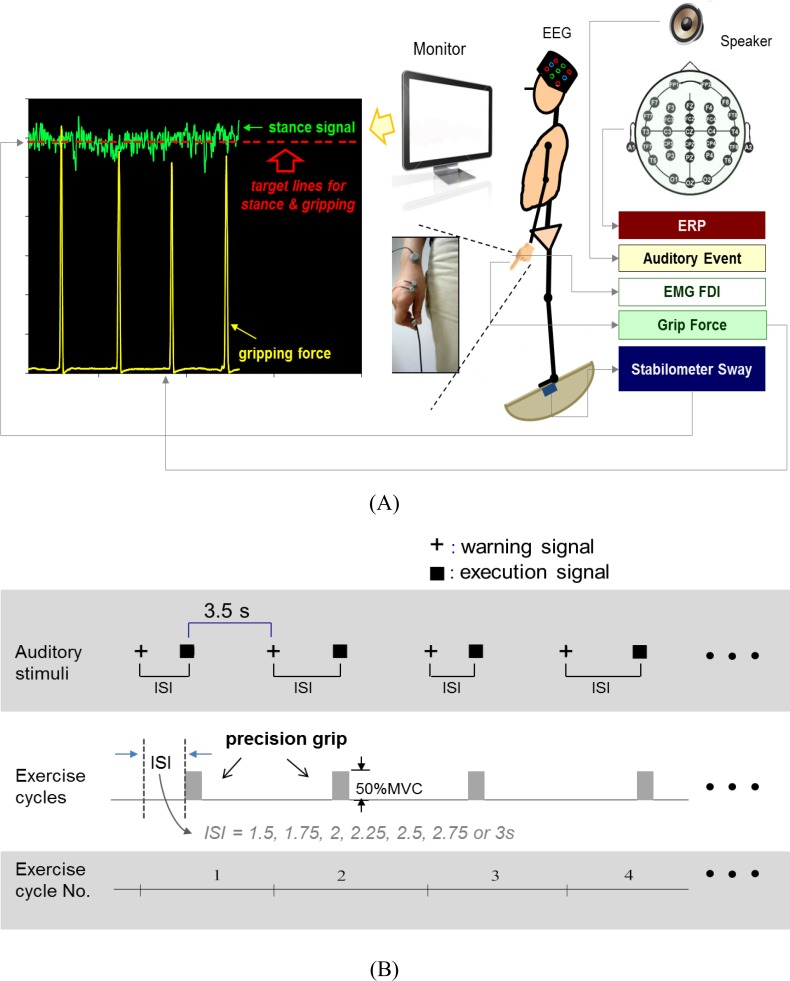
Diagram of study design. (A) Diagram of experimental setup and recordings of physiological data. (B) A schematic illustration of the auditory stimulus paradigm for the dual task (concurrent postural and force-matching tasks). Warning signals (+) to catch the subject’s attention were presented before execution signals (■), at which the subjects started a precision grip for force matching. The interval between the warning and the execution signals, or inter-stimulus interval (ISI), was randomized. A fixed interval of 3.5 seconds separated the execution signal and the next warning signal.

Before the experiment, we collected base-line information about the maximum voluntary contraction (MVC) of the right thumb-index precision grip and the maximal anterior tilt angle during stabilometer stance for each subject. There were two experimental conditions (single-posture vs. dual-task) for each participant. In the single-posture condition, the subjects were directed to stand on a stabilometer (a wooden platform (67 cm × 50 cm) with a curved base (height: 24 cm)) at 50% of the maximal anterior tilt. On-line visual information about the incline of the stabilometer was provided on an 18.5-inch monitor positioned at eye-level, 50 cm in front of the subjects. Subjects could remedy deviations of the stabilometer movement from the target angle at all times to maintain posture precision. In the dual-task condition, subjects were requested to perform a force-matching task and maintain their balance concurrently as the single-posture task (Fig 1A). For the force-matching subtask, the subjects executed a thumb-index precision grip to couple the target line of 50% MVC force instantaneously in response to auditory cues. Since this study aimed to exemplify how the brain worked to adapt to an additional motor task without causing postural destabilization (see discussion 4.4), the unique postural-motor setup was empirically determined in a preliminary study to limit repetitive force-matching attempts at large exertion levels that could cause motor fatigue during the experiment. The auditory cues consisted of 80-second sequences of tone pips in a total of fourteen warning-execution signal pairs (Fig 1B). To minimize the chance of participants predicting the force-matching execution signal after receiving a warning tone, the warning tone (an 800 Hz tone lasting for 100 ms) was randomly presented at different intervals of 1.5, 1.75, 2, 2.25, 2.5, 2.75 or 3 seconds before an execution tone (a 500 Hz tone lasting for 100 ms). The interval between the end of the execution tone and the beginning of the next warning tone was 3.5 seconds. Upon hearing the execution tone, the participants started a quick thumb-index precision grip (force impulse duration < 0.5 sec) to couple the peak precision-grip force with the force target on the monitor. In the dual-task condition, we strategically leveled the target signals for force-matching and posture in the same vertical position of the monitor to reduce the visual load during the concurrent tasking. There were six trials for the dual-task condition, with each trial composed of 14 precision grips. For the single-posture condition, subjects repeated the posture subtask as the dual-task condition but did not exert precision-grip force in response to auditory beeps and simply held the force-matching apparatus. Each participant was tested in a random order across experimental conditions.

### System set-up and data recording

The behavioral data of the posture and force-matching subtasks were measured. An inclinometer (Model FAS-A, MicroStrain, USA) was mounted on the center of the stabilometer to measure the tilting angle of the stabilometer. The level of force-matching was recorded with a load cell (15-mm diameter × 10-mm thickness, net weight = 7 grams; Model: LCS, Nippon Tokushu Sokki Co., Japan) mounted on the right thumb. The load cell was connected to a distribution box by a thin wire that could not provide stable mechanical support for the postural stance via the grip force apparatus. The activation of the first dorsal interosseous (FDI) muscle was monitored with a surface electromyogram (EMG) in a bipolar arrangement (Ag/AgCl, 1.1 cm in diameter, Model: F-E9-40-5, GRASS, USA) and an AC amplifier (gain: 500, cut-off frequency: 1 and 300 Hz; Model: P511 series, GRASS, USA). The auditory stimuli and target signals for conducting force-matching and posture subtasks were generated with LabVIEW software (National Instruments, Austin, TX, USA). Thirty-two Ag-AgCl scalp electrodes (Fp_1/2_, F_z_, F_3/4_, F_7/8_, FT_7/8_, FC_z_, FC_3/4_, FC_7/8_, C_z_, C_3/4_, CP_z_, CP_3/4_, P_z_, P_3/4_, T_3/4_, T_5/6_, TP_7/8_, O_z,_ and O_1/2_) with a NuAmps amplifier (NeuroScan Inc., EI Paso, TX, USA) were used to register scalp voltage fluctuations in accordance with the extended 10–20 system. The ground electrode was placed along the midline ahead of F_z_. Electrodes placed above the arch of the left eyebrow and below the eye were used to monitor eye movements and blinks. The impedances of all the electrodes were below 5 kΩ and were referenced to linked mastoids of both sides. All physiological data were synchronized and digitized at a sample rate of 1 kHz.

### Data analyses

#### Behavior data

The behavior parameter of the force-matching subtask in the dual-task condition was assessed with normalized force-matching error (NFE), which was denoted as $\frac{\left|PGF-TF\right|}{TF}\times 100\%$ (where PGF: peak grip force; TF: target force). The NFE of all force-matching events was averaged across trials for each subject in the dual-task condition. The behavior parameters of the posture subtask included the size and regularity of posture error, defined as the mismatch between the execution tone and the timing of force pulse initiation (movement onset). The inclinometer data were first conditioned with a zero-phase low-pass filter (cut-off frequency: 6 Hz), followed by linear transformation to degrees out of balance. The size and regularity of posture error were determined by application of the root mean square (RMS) and sample entropy (SampEn) on the posture error. SampEn provides a regularity index of postural sway [7,8]. It ranges from 0 to 2, with the larger value representing more complexity. Before the calculation of SampEn, the trajectories of posture error signal were first normalized with the standard deviation of the time series. SampEn measured the negative natural logarithm of an estimate of the conditional probability that epochs of length m that matched point-wise within a tolerance level (*r*) also matched at the next point for a time series data with a total data point number of *N*. The mathematical formula for SampEn was

$$SampEn(m,r,N)=\text{ln}\left(\frac{\sum_{i=1}^{N-m}n_{i}^{m}}{\sum_{i=1}^{N-m-1}n_{i}^{m+1}}\right)=\text{ln}(\frac{n_{n}}{n_{d}})$$

where *r* = 15% of standard deviation of the error signal and *m* = 3.

#### ERP parameterization and temporal-spatial analyses

The DC shift of each channel was compensated for off-line analysis using a third-order trend correction over the entire set of recorded data. The continuous EEG data were conditioned with a low pass filter (40 Hz/48 dB roll-off) and segmented into epochs of 700 ms, including 100 ms before the onset of each execution signal (all ERPs were aligned to the execution signal). Each epoch was visually inspected, and those with artifacts (such as excessive drift, eye movements, or blinks) were removed using the NeuroScan’s 4.3 software (NeuroScan Inc., EI Paso, TX, USA). Only epochs with proper responses were averaged (at least 75 trials for each experimental condition) following baseline-correction at the pre-stimulus interval. Mean amplitudes within the 80–150 and 150–240 ms time windows were used to quantify the amplitudes of N1 and P2, which are modifiable to manage posture and perceptual-motor supraposture events, respectively [20,21]. In addition, because frontal and parietal cortices involved in coordination of limited central resource and shifting of attention in dual-tasking [29,30], we applied Global Field Power (GFP) computation to ERP of the frontal (F_z_, F_3_, F_4_, FC_z_, FC_3_, and FC_4_) and sensorimotor-parietal areas (C_z_, C_3_, C_4_, CP_z_, CP_3_, CP_4_, P_z_, P_3_, and P_4_), respectively. GFP was used to measure ERP response strength and the resultant GFP waveform is a measure of potential (uV) as a function of time. With a measure of the spatial standard deviation at a given time [31–33], GFP quantifies the integrated electrical activity for each electrode in the regions of interests and also can prevent multiple comparisons among electrodes. The resultant GFP waveform allowed us to determine the peak magnitude of the integrated aspects of the N1 and P2 components in the frontal and sensorimotor-parietal areas. In addition, the Global Map Differences (GMD) [32,33] was calculated to compare ERP topographies in the frontal and sensorimotor-parietal areas for a given time slot between the two experimental conditions. The resulting index of the GMD, topologic dissimilarity (DISS), was calculated for testing topography homogeneity between two electric fields. DISS is independent of the strength of electric fields and ranges from 0 (topologic homogeneity) to 2 (topologic inversion). The mathematical formula for GFP and DISS was

$$\bar{u}=\frac{1}{n}\times \sum_{i=1}^{n}U_{i}$$

$$u_{i}=U_{i}-\bar{u}$$

$$GFP_{u}=\sqrt{\frac{1}{n}\times \sum_{i=1}^{n}u_{i}^{2}}$$

$$DISS_{u,v}=\sqrt{\frac{1}{n}\times \sum_{i=1}^{n}{\left(\frac{u_{i}}{GFP_{u}}-\frac{v_{i}}{GFP_{v}}\right)}^{2}}$$

where *n* is the number of electrodes of each interested area; *U*_*i*_ is the measured potential of the *i*th electrode, for a given condition *U*, at a given time point t; and *V*_*i*_ is the measured potential of the *i*th electrode from another condition *V* [32].

#### ERP principal component analysis and time-frequency distribution

Prior to ERP spectral analysis, principal component analysis (PCA) was performed on the ERP of each subject in the frontal and sensorimotor-parietal areas. Spatial PCA with a covariance method was used to condense high dimensional across-trial averaging ERP by transforming multivariate grand-averaged ERP data into a major ERP principal component (PC1). Frontal PC1 and sensorimotor-parietal PC1 could sufficiently account for the phase-locking ERP in reference to the execution signal. This study contrasted the spectral features of the PC1s between single-posture and dual-task conditions, and within each context, inferred unique PC1 time-frequency distributions due to the addition of a secondary motor task on stabilometer stance. The spectral responses of PC1 were computed via a wavelet-based time-frequency analysis [34,35]. The PC1 temporal profile of each subject was convolved with complex Morlet wavelets to yield the wavelet transform *W*(*t*, *f*):

$$W(t,f)=\frac{1}{\sqrt{\left|a\right|}}\int_{-\infty }^{\infty }s(t)\varphi^{*}(\frac{t-b}{a})dt$$

where φ*($\frac{t-b}{a}$) is the complex conjugate of Morlet mother wavelet, *a* is the scale factor, *t* is the time translation, and *s*(*t*) is the signal of interest (PC1 temporal profile).

The wavelet transform was performed over a 500 ms interval from 100 ms pre- to 400 ms post-execution signals, including N1 and P2 in the frontal PC1 and sensorimotor-parietal PC1 for the posture and dual-task conditions. Wavelet analysis on averaged ERP principle components could only feature phase-locked components within ERP activities [36]. To this end, we examined the significance of the spatiotemporal pattern of the ERP time-frequency distribution of the frequency bands that showed prominent activity (delta (1–4 Hz), theta (4–8 Hz), and beta (13–35 Hz)), by representing differences in the normalized wavelet transform between the single-posture and dual-task conditions with Z values on a basis of a paired difference test:

$$Z(t,f)=\frac{(\bar{W_{Dual}(t,f)}-\bar{W_{Single}(t,f))}}{S_{d}(t,f)/\sqrt{n}}$$

where $\bar{W_{Dual}(t,f)}$ is the mean values of the normalized ERP wavelet transform in the dual-task condition; $\bar{W_{Single}(t,f)}$ is the mean value of the normalized ERP wavelet transform in the single-posture condition; *S*_*d*_*(t*,*f)* is the pooled standard deviation of the normalized differences between all pairs of $\bar{W_{Dual}(t,f)}$ and $\bar{W_{Single}(t,f)}$; and n is the number of subjects. For the single-posture and dual-task conditions, the time-dependent spectral coupling of the frontal and sensorimotor-parietal areas was characterized by applying the same wavelet analysis on cross-correlation between the frontal PC1 and the sensorimotor-parietal PC1. Likewise, differences in the normalized dynamic cross-spectra (coherence) between the two PC1s were represented with Z values based on a paired difference test. The coherence is a normalized measure of coupling between two signals at any given frequency, and it varies between 0 (no correlation) and 1 (perfect correlation). In the present study, the coherence between the frontal ERP PC1 and the sensorimotor-parietal ERP PC1 was calculated by using the formula [37,38]:

$$Coh_{xy}\left(\lambda \right)={\left|R_{xy}\left(\lambda \right)\right|}^{2}=\frac{{\left|f_{xy}\left(\lambda \right)\right|}^{2}}{f_{xx}\left(\lambda \right)f_{yy}\left(\lambda \right)}$$

In this equation, *f* characterizes the special estimate of two EEG signals *x* (frontal ERP PC1) and *y* (sensorimotor-parietal ERP PC1) for a given frequency (λ). The numerator includes the cross-spectrum for *x* and *y* (*f*_*xy*_), whereas the denominator includes the autospectra for x (*f*_*xx*_) and y (*f*_*yy*_).

### Statistical analysis

The paired-t statistic was used to examine the differences in postural performance variables, including RMS and SampEn on the posture error (Err_RMS and Err_SampEn), and ERP amplitudes (peak magnitude of N1 and P2 in a GFP waveform) between the single-posture and dual-task conditions. Similarly, the paired-t test was also used to examine differences in the wavelet transform elements of ERP principal components between the two experimental conditions. To compare scalp ERP topographies in the frontal and sensorimotor-parietal areas between single-posture and dual-task conditions, topological ANOVA (or TANOVA [33,39]) was used (see [32] for further details). Based on 5,000 permutations, the statistical approach is a non-parametric bootstrapping procedure for estimating the probability of task-dependent DISS results from each time point at a within-subject level. The calculated value of TANOVA, is “1 –p value”; therefore, while the TANOVA value is larger than 0.95, the p value is smaller than 0.05, indicating there is a significant DISS difference between the two maps [32]. The level of significance was set at *p* = 0.05. Signal processing of the behavior data and statistical analyses were performed in Matlab (Mathworks Inc. Natick, MA, USA) and the statistical package for SPSS statistics v. 17.0 (SPSS Inc. Chicago, IL, USA). All data were represented with mean ± standard error.

## Results

### Behavior results

The effect of the additional motor task on postural performance was assessed with changes in the size and complexity of postural error prior to the movement onset. The results of paired-t statistics revealed an insignificant effect of added force-matching on the Err_RMS (single-posture: 0.159 ± 0.014 degree; Dual-task: 0.165 ± 0.015 degree)(*t*_*11*_ = —.931, *p* = 0.370). In contrast, the dual-task condition exhibited a significantly larger Err_SampEn than the single-posture condition (single-posture: 0.393 ± 0.017; dual-task: 0.411 ± 0.018)(*t*_*11*_ = - 3.776, *p* = 0.003). Normalized force-matching error in the dual-task condition was 10.26 ± 0.90%.

### N1 and P2 Components of ERPs

**Fig 2:**
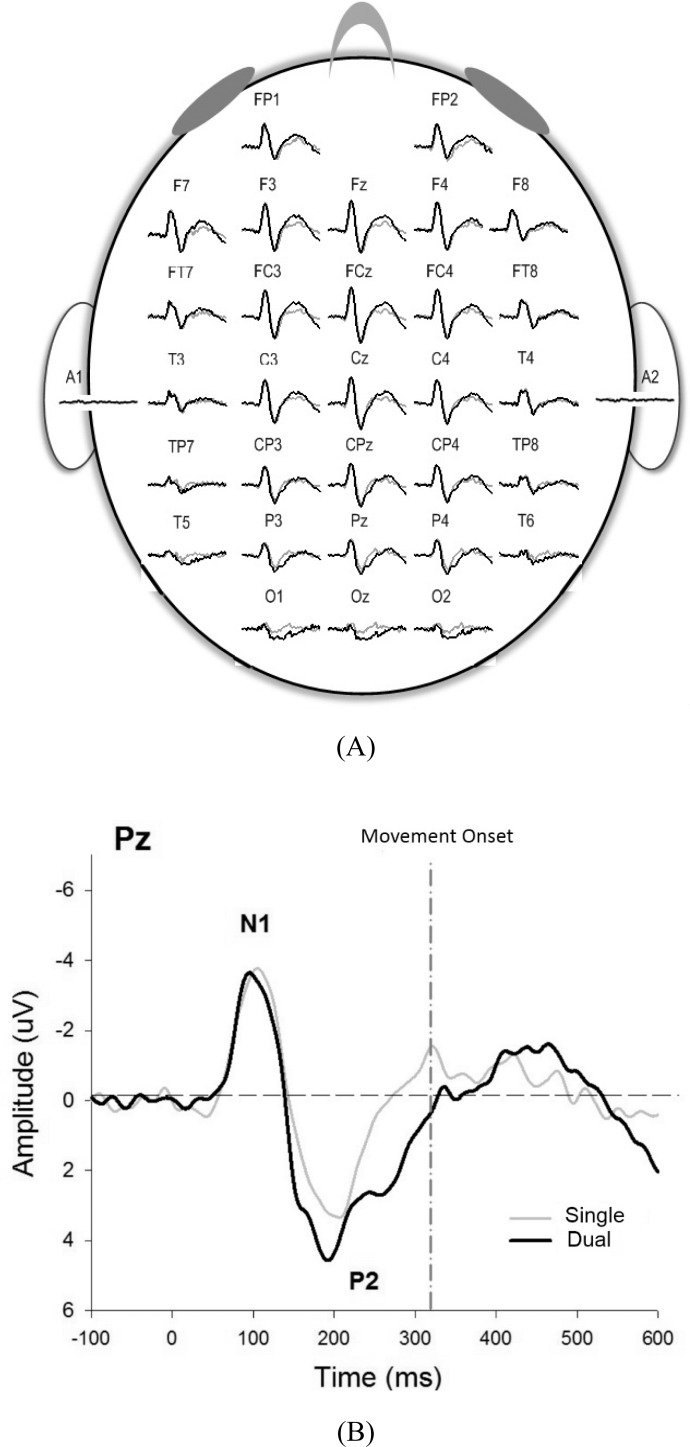
Plots of grand-average ERPs. (A) Grand-average ERPs recorded from all 12 subjects in the single-posture and dual-task conditions. (B) A grand average ERP waveform of the Pz electrode with two major ERP components (N1 and P2) before force-matching in the dual-task condition. The characteristic changes in N1 and P2 between components reflects the cognitive cost of the added force-matching superimposed on a stabilomater stance (grey line: single-posture condition; black line: dual-task condition).

**Fig 3:**
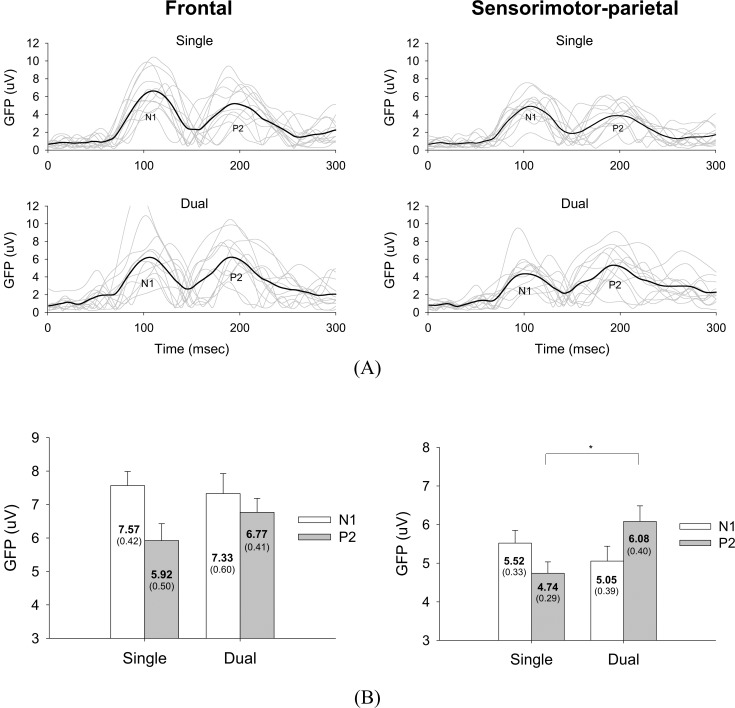
Global field power (GFP) of the single-posture and dual-task conditions. (A) Temporal profiles of GFP for all subjects. Black-bold lines indicate subject-averaged GFP profiles (n = 12). (B) The contrasts of peak GFP of the N1 and P2 components between the single-posture and dual-task conditions (left plot: frontal area; right plot: sensorimotor-parietal area)(*: *p* < .05).

Fig 2 shows the population means of all the scalp-recorded ERP and pooled ERP profiles at the P_z_ for the single-posture and dual-task conditions. We restricted our observations of ERP responses in the frontal and sensorimotor-parietal areas to the repeated presentations, reasoning that the additional motor-suprapostural task on top of postural control would reveal primary brain mechanisms of cooperative activities within the areas. In terms of GFP function, Fig 3A illustrates field strength modulation in the frontal and sensorimotor-parietal areas for the single-posture and dual-task conditions. A paired-t test revealed that the peak GFP of the N1 and P2 components in the frontal area did not differ between the two task conditions (Fig 3B, left plot)(N1: *t*_*11*_ = 0.418, *p* = 0.684; P2: *t*_*11*_ = -1.326, *p* = 0.212). In the sensorimotor-parietal area, the peak GFP value in the P2 period was paradigm-dependent, with a greater peak P2 GFP for the dual-task condition (N1: *t*_*11*_ = 0.874, *p* = 0.401; P2: *t*_*11*_ = -2.688, *p* = 0.021).

**Fig 4:**
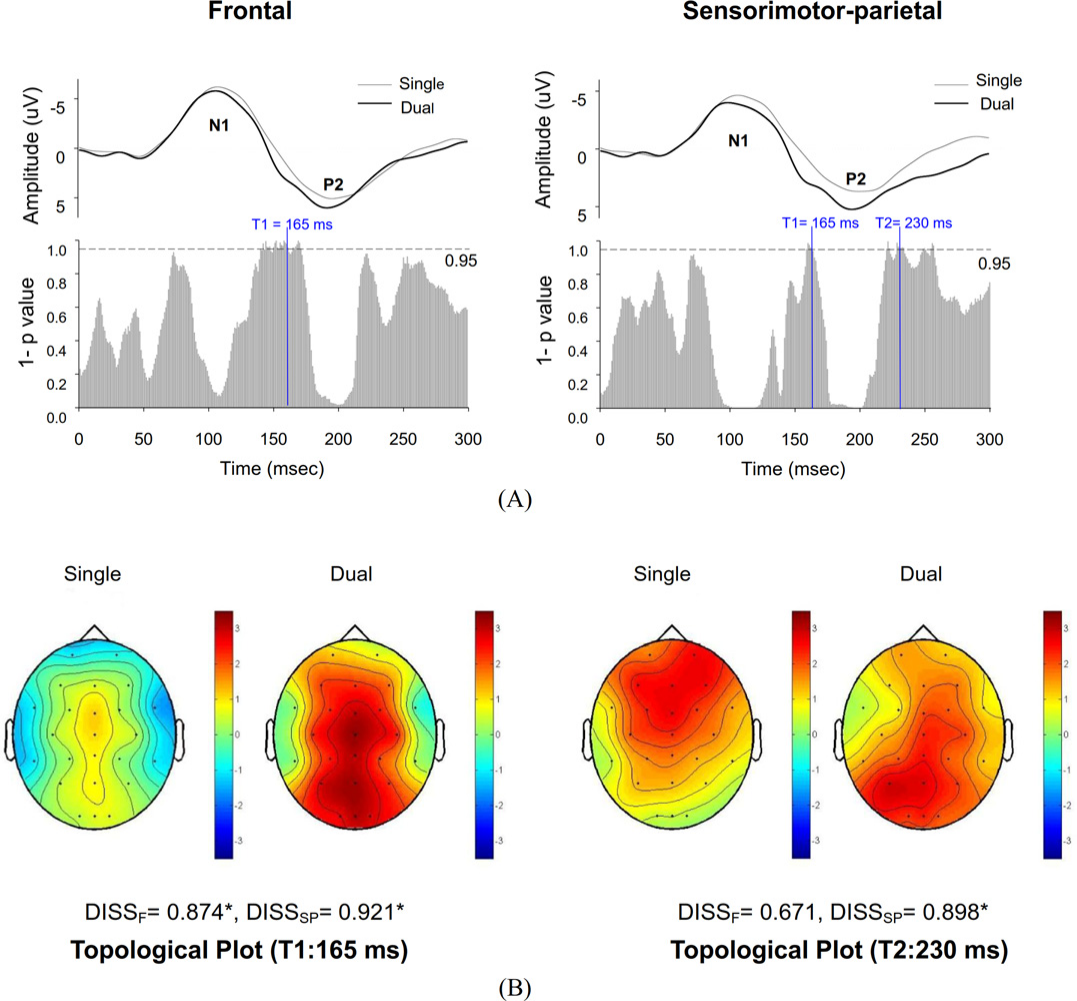
Topological ERP analysis of the single-posture and dual-task conditions. (A) Millisecond-by-millisecond topological ANOVA (TANOVA) results. T1 is a time point that exhibits ERP topological differences for the frontal and sensorimotor-parietal areas between the single-posture and dual-task conditions. T2 is a time point that only exhibits ERP topological differences in the sensorimotor-parietal areas between the two conditions. (B) Scalp topologies and global dissimilarity (DISS) at T1 and T2 for the single-posture and dual-task conditions. (*: *p* < 0.05).

Fig 4 displays spatial statistics and scalp topographies of ERP for the single-posture and dual-task conditions, in light of global dissimilarities of the frontal and sensorimotor-parietal areas between these two conditions. Based on a Monte Carlo bootstrapping approach, millisecond-by-millisecond TANOVA revealed significant topographic modulations of frontal ERP around 140–170 ms, a transitional phase of the N1 and P2 period (Fig 4A, left plot). In addition, TANOVA statistics indicated significant topographic modulations of sensorimotor-parietal ERP around 165 ms and 230 ms (Fig 4A, right plot). Fig 4B displays pooled ERP scalp topographies at two representative time points (165 ms and 230 ms) when a configuration difference in ERPs due to the added motor-suprapostural task was evident. At the N1-P2 transitional phase (T1:165ms), the power of the electrical field in the frontal (DISS_F_ = 0.874, *p* < .05) and sensorimotor-parietal (DISS_SP_ = 0.921, *p* < .05) areas was more pronounced in the dual-task condition than in the single-posture condition. At T2 (235 ms), it was clear that the dual-task condition had more P2 activity in the sensorimotor-parietal area (DISS_SP_ = 0.898, *p* < .05) than the single-posture condition, which instead caused more P2 activity in the frontal areas.

**Fig 5:**
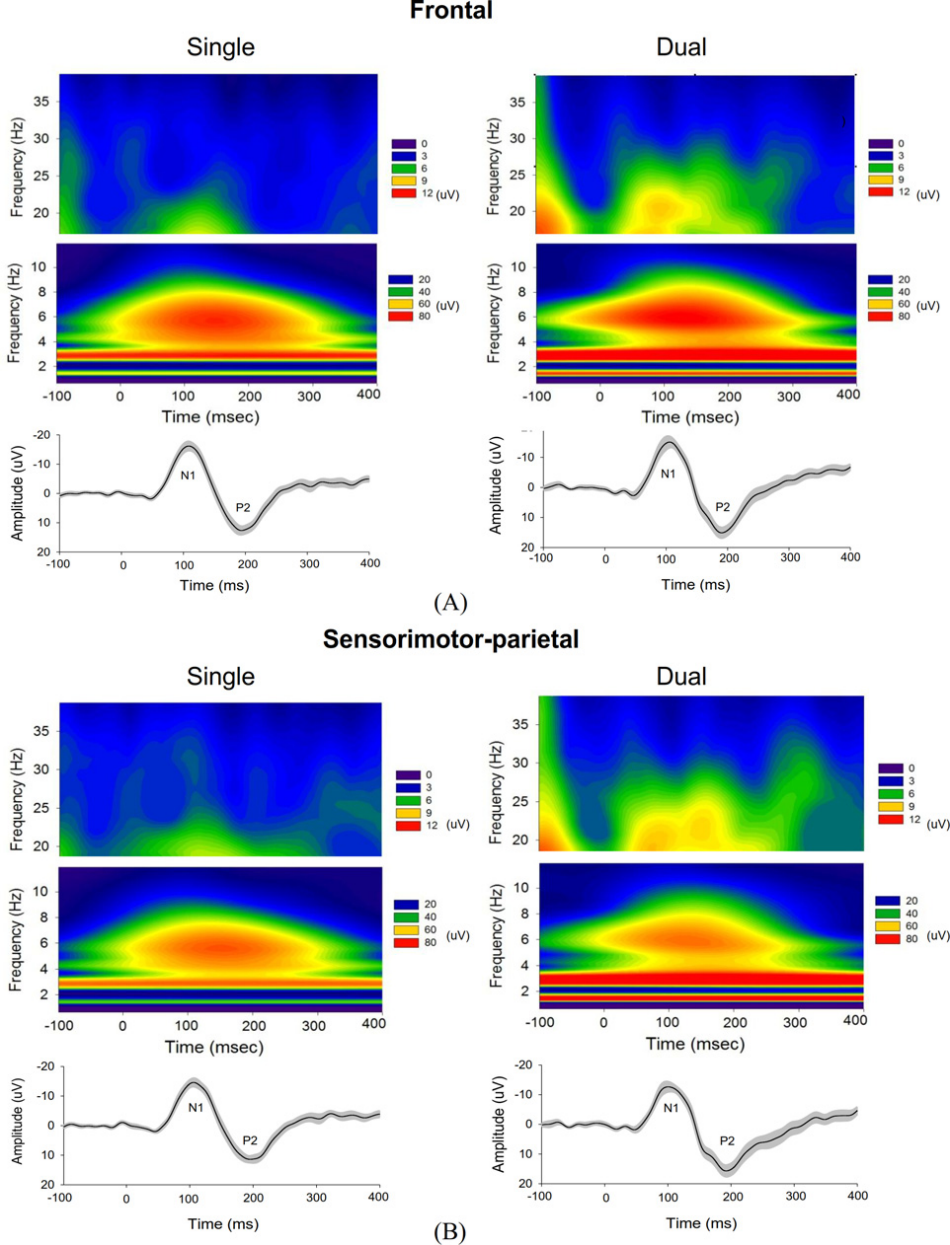
Principal components (PC) of the ERP for the single-posture and dual-task conditions. (A) and (B) represent ERP PC analysis in the frontal and sensorimotor-parietal areas, respectively. The upper and middle rows represent the pooled time-frequency distribution of PC1 (the primary principal component of ERP in the regions of interest) in the 13–35 Hz and 1–12 Hz spectral bands, respectively. The bottom row is the mean temporal profile of ERP PC1, which clearly exhibits N1 and P2 components.

**Fig 6:**
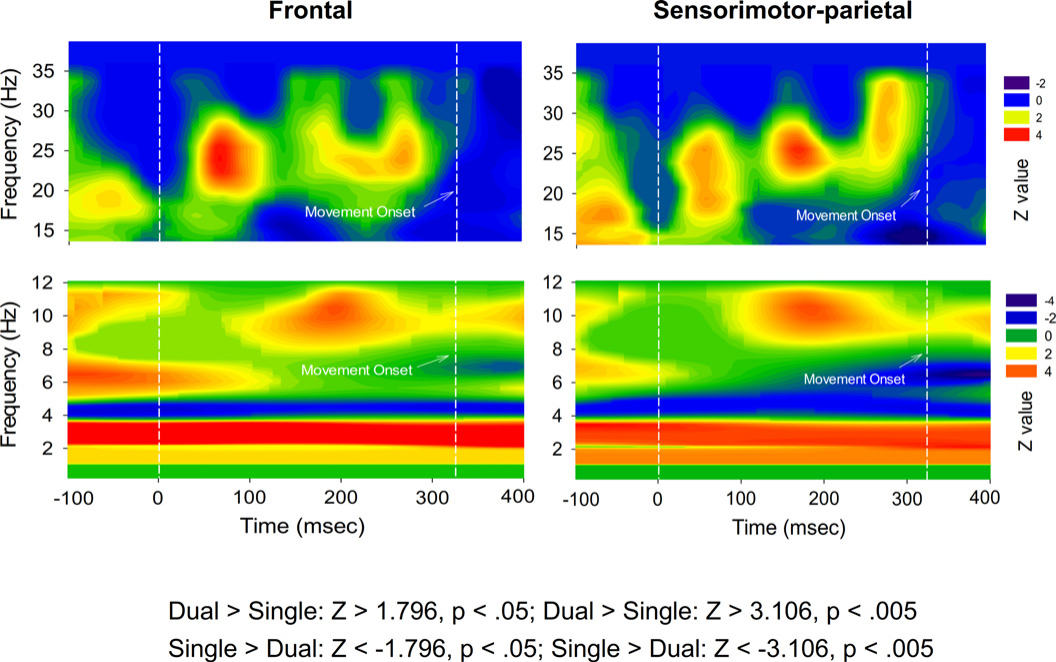
Normalized task-related differences in time-frequency distribution for the PC1 in terms of Z values. The dual-task condition exhibited 1) a greater persistent delta oscillation in the frontal and sensorimotor-parietal areas; 2) persistent suppression of theta oscillation in the frontal and sensorimotor-parietal areas; and 3) a more pronounced beta (13–35 Hz) synchronization around 100 ms and 200 ms after the executive signals. (Although only the dual-task condition has movement onset value for the force-matching task, we also labelled the lines in the Figs of the single-posture condition for the ease of comparison with those of the dual-task condition).

**Fig 7:**
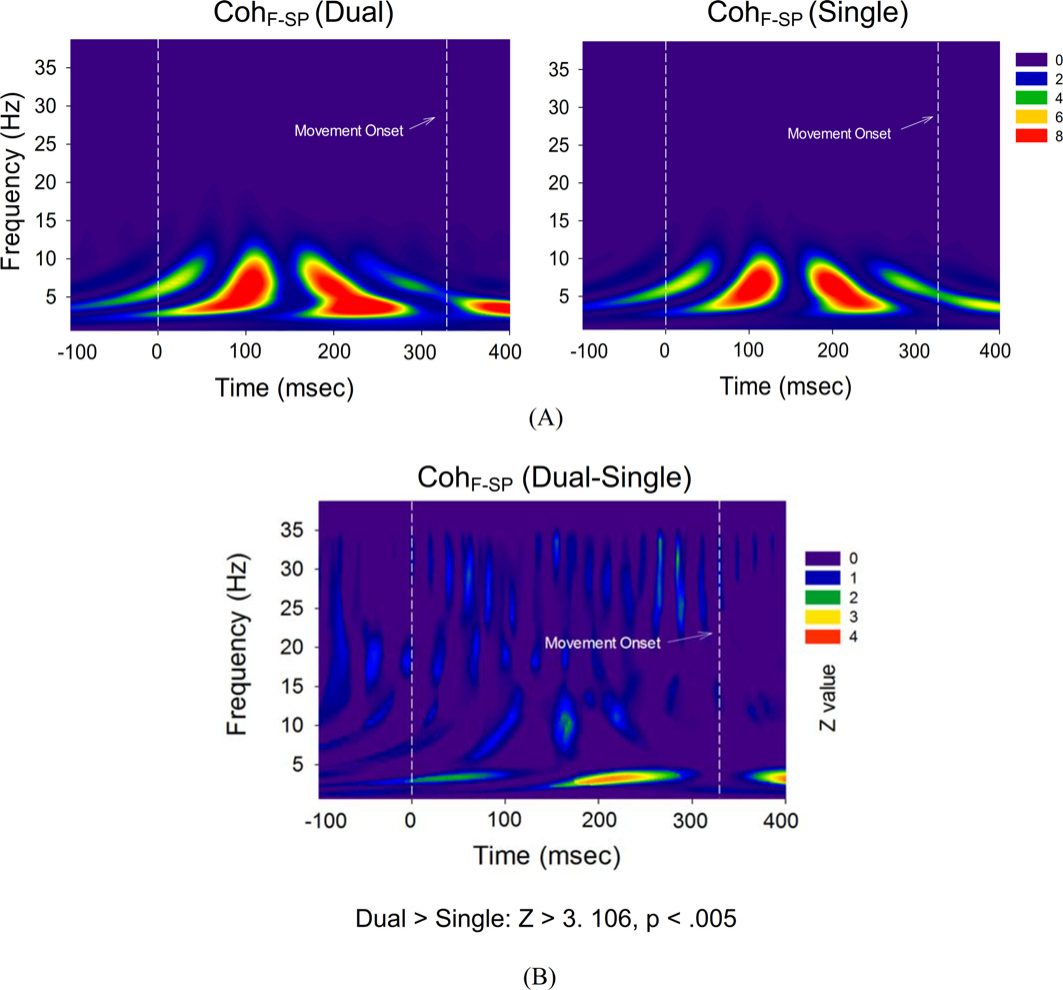
Time-dependent cross-spectra of PC1. (A) Time-dependent cross-spectra between PC1s in the frontal and sensorimotor-parietal areas in the single-posture and dual-task conditions. (B) Normalized task-related differences in time-dependent PC1 cross-spectra in terms of Z values. The dual-task condition exhibited stronger delta coherence between the frontal and sensorimotor-parietal areas in the P2 period (*p* < .005).

To assess the spectral features of ERP, high dimensional ERP in the frontal and sensorimotor-parietal cortex was represented with the first principal component (PC1) for both executive conditions. Frontal PC1 accounted spatially for more than 90% of the ERP variance properties of the regions of interest (single-posture: 93.2 ± 0.9%; dual-task: 92.1 ± 0.7%). PC1 of the sensorimotor-parietal area also accounted sufficiently for the ERP variance properties of the regions of interest (single-posture: 94.2 ± 1.0%; dual-task: 92.9 ± 0.7%). This fact validated the use of PC1 to represent the time-frequency analysis across numerous electrodes. Fig 5A shows the mean frontal PC1 profile and pooled spectral power map of frontal PC1 for the single-posture and dual-task conditions. The temporal profiles of frontal PC1 contained discernible N1 and P2 components, resembling the scalp-recorded ERP waveforms. Three discernible particular EEG oscillations, the delta (1–4 Hz), theta (4–8 Hz), and beta (13–35 Hz) rhythms, were noted for both conditions. Similar temporal and spectral characteristics were found in the PC1 of the sensorimotor-parietal area for both the single-posture and the dual-task conditions (Fig 5B). The delta activity of the frontal and sensorimotor-parietal PC1s that presented throughout the recording period was not phase-dependent, whereas the theta and beta rhythms were modulated around the N1-P2 period. The difference in PC1 energy of the time-frequency distribution between the two experimental conditions was represented in terms of Z values (Fig 6). For frontal and sensorimotor-parietal PC1, major characteristic changes in the dynamic spectra due to the addition of a motor-suprapostural task were persistent delta potentiation/theta suppression and phasic beta synchronization in the N1 and P2 periods in the dual-task condition. Fig 7A contrasts time-dependent cross-spectra of the frontal PC1 and the sensorimotor-parietal PC1 between the single-posture and dual-task conditions. The impact of the motor-suprapostural task on the fronto-sensorimotor-parietal interplay in the spectral domain was phase-dependent, including enhanced delta coherent activity in the P2 period (180–280 ms) for the dual-task condition (Fig 7B).

## Discussion

### Dual-task effect on postural performance

Despite an insignificant change in the size of posture error during stabilometer stance, the behavioral costs of an added motor-suprapostural task were increased in the sample entropy of posture errors before force-matching. The increase in irregularity of postural performance showed similarities to previous dual-task studies, which used a cognitive-suprapostural task with the eyes closed [7] and a challenging cognitive/motor suprapostural tasks in a dual-task setup [40,41]. Postural regularity was noted to positively correlate with amount of attention allocated to postural control. The higher postural irregularity (or higher entropy of postural sway) indicates a more automatic responses, as the less attentional resource is devoted to postural control and correlates with a more efficient and automatic of balance maintenance [7,8,40]. Hence, the increase in postural irregularity in the dual-task condition might well be hypothesized to be an increase in postural automaticity (or less attentional investment to the posture subtask) due to the addition of a secondary motor task. The results of force-matching error in the dual-task condition (force-matching from the tilted stabilometer board)(NFE: 10.26 ± 0.87%) indicated that the participants did not give up force-matching during the experiment, as compared to the NFE of force-matching from a level surface (NFE: 9.85 ± 0.77%)(data from the pilot study; *t*_*22*_ = 1.259, *p* = 0.22 with Student’s t test). A more autonomous postural control in the dual-task condition could be advantageous to share attentional resource with task-shifting and planning of secondary force-matching task. However, behavior phenomena were limited to lend insight into the brain mechanisms for postural-motor interaction in the dual-task condition.

### Dual-task effect on temporal-spatial patterns of ERP

The major differences in the temporo-spatial feature of ERP between the single-posture and dual-task conditions were 1) enhanced P2 positivity in the bilateral sensorimotor-parietal area (Fig 3) and 2) marked topographic modulation of ERPs in the N1-P2 transitional phase and late P2 component (Fig 4). In accordance with the serial order of processes and the “posture-first” principle [42], previous studies have shown that the earlier N1 component of a postural-suprapostural task reflects anticipatory arousal [23] and sensory processing for postural perturbation [24,25], whereas positivity of the latter P2 component is a function of task difficulty [20] and attentional focus [21] of the secondary force-matching task. Concerning the preceding results, the similar size of the postural sway in the single-posture and dual-task conditions is partly explained by task-invariant modulation of the amplitude of N1. On the other hand, the enhanced positivity of P2 in the sensorimotor-parietal area (Fig 3B) was a direct consequence of the increase in cognitive load during execution of the added motor-suprapostural task. Of particular interest in this study is the heuristic evaluation of the topographic modulations of ERP, which specified a more widespread P2 activation in the frontal or sensorimotor-parietal network early, at around 150 ms, and in the sensorimotor-parietal network around 230 ms after the execution beep (Fig 4). Namely, an added motor-suprapostural task taxed more of the central resources during the N1-P2 transitional phase and the late P2 period. Attributable to a positive wave of slow onset through frontal-parietal interaction [43], P2 modulation at around 150 ms in the frontal and sensorimotor-parietal areas could reflect a task-shift cost from the posture subtask to the force-matching subtask, cognitively in relation to time estimation in the paradigm switch [44–47] or filtering out irrelevant task information for the ease of decision making [43]. In contrast to frontal emphasis in the single-posture condition (Fig 4B), the latter prevailing P2 activity in the sensorimotor-parietal network at around 230 ms of the dual-task condition might be linked to integration of perceptual and executive processes preceding a goal-directed action [48–50]. This P2 activity could be related to the capture of target information by location and the selection of a response for force scaling in reference to the perceived target movement [50]. Because the resource capacity is a function of the activated area and the duration of activation of the brain [51], timely expansion of central resources in the dual-task condition was helpful to alleviate resource competition between the postural and suprapostural tasks (or bottleneck interference [52]), by increasing the alternative use and task-specific allocation of a central resource for performing a visuomotor dual-task [53]. Overall, our ERP results supported a situation-dependent tradeoff phenomenon [28] for concurrent postural and motor-suprapostural tasks.

### Task-specific modulation of PC spectral dynamics

In addition to the temporal-spatial patterns, our study revealed task-related frequency modulations of the ERP responses (Fig 5), which implies alterations in cognitive processes using different cortical cell assemblies. Particularly worth of note are the enhancement of delta (1–4 Hz) and early suppression theta (4–8 Hz) powers (Fig 6) due to the addition of a motor-suprapostural task. First, functionally gated by the cingulate pathway, a sustained facilitation of delta powers in the frontal and sensorimotor-parietal areas in the dual-task condition could be the propagation of delta oscillation along the posterior axis of the brain, hypothesized to be linked with motivationally-relevant states [54]. Hence, we speculated that the global potentiation of ERP delta power could index motivational enhancement after the goal setting of a secondary motor task in a dual-task situation. Of specific interests was the delta coherent activity in the frontal and sensorimotor-parietal areas, which was time-locked to the supraposture-dependent P2 component (~200 ms)(Fig 7B). Conceptually in agreement with previous knowledge gained using the Go/No-Go paradigms [55–58], the coherent delta activity could be a means of quality control of the force-matching subtask by temporarily excluding functionally-irrelevant events from force-matching execution, such as ongoing multimodal sensory inputs from postural systems. Besides, the enhanced delta coherence indicated a greater dual-task cost of interesting information transfer between frontal and sensorimotor-parietal areas [57]. Next, human verticalization revealed a specific increase in the EEG theta oscillatory activity in the fronto-central and occipito-parietal regions [59,60]. Also, theta strength is a function of postural vigilance [61], granting that theta power during upright stance becomes stronger due to unpredictable perturbation [62,63]. Hence, the progressive waning of theta synchrony in the frontal and sensorimotor-parietal areas in this study indicates a reduction in attentional control in the posture subtask [64,65], in preparation for a response shift to the subsequent suprapostural task in the dual-task condition. Finally, beta oscillations were sporadically potentiated with the added motor-suprapostural task (Fig 6). Increases in interregional interactions in the beta band have also been reported in dual-tasks with a verbal-manual or visual perceptual-visual spatial working memory setup as compared to those in a single-task condition [19,66]. The enhanced beta activity highlights the increased neural communication of executive functions to process a vast amount of information.

### Methodological concerns

This novel work is a preliminary study to investigate neural correlates of task switching and task cost for stance control with additional motor task. Our findings should be generalized very cautiously to other posture-motor situations. First, central resource allocation could vary with the relative task load of a posture-motor dual-task, as constraints placed on postural control by suprapostural task goals are divergent, depending on task difficulty, according to studies of behavior results [67,68]. By manipulation of force-matching among 25%, 50% and 75% of MVC, our pilot study (n = 12; subjects in the pilot study were different from those in the main experiment) using the current experimental setup revealed that the postural characteristics did not vary with the force-exertion level (p > 0.05), whereas force-matching at 50% MVC exhibited satisfactory within-subject consistency without causing motor fatigue. For the sake of convenience, we investigated brain mechanisms under the condition of a target force of 50% of MVC in this study. To investigate the effect of the relative task load on the neural cost of dual-task interference, undoubtedly, further investigation need to consider all combinations of exertion levels of force-matching and tilting angles of stabilometer stance. However, that is beyond the scope of this study. Second, wavelet analysis was performed on averaged ERP principle components in this study. The methodological merit of principal component analysis is the effective reduction of dimensions, at the cost of underestimation of the non-phase locked spectral components. Although the formalism of the time-frequency transform is restricted to the phase-locked spectral components of ERP, the non-phase locked spectral components following the PCA process are not physiologically interpretable. The reason is that the asynchronous neural activities with latency jitters from the ERP responses of multiple recording sites were differentially weighted. On account of the wealth of information available based on phase-locked ERP responses, it is recommended that further research specify the contribution of the non-phase locked spectral components to neural control of a supraposture subtask and the interplay between posture and supraposture subtasks.

## Conclusion

Using integrated analysis of phase-locked electrocortical signals, this study is the first to reveal the neural cost of the addition of a secondary motor-suprapostural task to a tilted stabilometer stance. Decrease in attentional control of posture task thanks to an added motor-suprapostural task was corroborated by postural irregularity, enhanced P2 positivity, and decreased ERP theta power in the dual-task condition. These task-specific alternations support resource reallocation of the brain relevant to a facilitatory role of postural control. The functional benefit is to exploit a more automatic strategy for effectively planning the secondary motor-suprapostural task in the dual-task condition.
